# “Appropriate Treatment” and Therapeutic Window in Spasticity Treatment with IncobotulinumtoxinA: From 100 to 1000 Units

**DOI:** 10.3390/toxins10040140

**Published:** 2018-03-28

**Authors:** Giancarlo Ianieri, Riccardo Marvulli, Giulia Alessia Gallo, Pietro Fiore, Marisa Megna

**Affiliations:** Department of Basic Sciences, Neuroscience and Sense Organs, University of Bari “Aldo Moro”, G. Cesare Place 11, 70125 Bari, Italy; ricmarv81@hotmail.it (R.M.); giulia.gallo1985@gmail.com (G.A.G.); p_fiore@hotmail.it (F.P.); marisa.megna@uniba.it (M.M.)

**Keywords:** spasticity, botulinum toxin type A, appropriate treatment, Therapeutic Index

## Abstract

Many neurological diseases (ischemic and hemorrhagic stroke, multiple sclerosis, infant cerebral palsy, spinal cord injuries, traumatic brain injury, and other cerebrovascular disorders) may cause muscle spasticity. Different therapeutic strategies have been proposed for the treatment of spasticity. One of the major treatments for tone modulation is botulinum toxin type A (BTX-A), performed in addition to other rehabilitation strategies based on individualized multidisciplinary programs aimed at achieving certain goals for each patient. Therapeutic plans must be precisely defined as they must balance the reduction of spastic hypertonia and retention of residual motor function. To perform and optimize the treatment, an accurate clinical and instrumental evaluation of spasticity is needed to determine how this symptom is invalidating and to choose the best doses, muscles and times of injection in each patient. We introduce an “appropriate treatment” and no “standard or high dosage treatment” concept based on our retrospective observational study on 120 patients lasting two years, according to the larger Therapeutic Index and Therapeutic Window of Incobotulinumtoxin A doses from 100 to 1000 units. We studied the efficiency and safety of this drug considering the clinical spasticity significance for specialist physicians and patients.

## 1. Introduction

Spasticity is the most common complication of many neurological diseases followed by ischemic and hemorrhagic stroke, multiple sclerosis, infant cerebral palsy, spinal cord injuries, traumatic brain injury, and other cerebrovascular disorders [1].

Comprehending the various mechanisms of muscle tone alteration and the quantitative evaluation of muscle rheological modifications can lead to the development of more precise and targeted therapeutic interventions for the treatment of spasticity [2,3]. Anatomically, there is a reduction in type II fibers and an increase in type I fibers. Clinically, it is an involuntary motor disorder, characterized by hypertonic muscle tone with increased excitability of the muscle relaxation reflex and increased tendon reflexes. Muscle weakness or paresis in the limbs is associated with spasticity and contributes to loss of motor dexterity and functional capacity [4]. Spasticity, if left untreated, can hinder the functional result by promoting persistent abnormal postures which produce muscle-tendon contractions and bone deformities. Substantial complications resulting from spasticity include movement impairment, difficulty in managing hygiene and self-care, poor self-esteem, body image alteration, pain, and pressure ulcers. In addition, patients with severe spasticity may suffer poor social interaction drastically worsening quality of life [5,6].

For these clinical problems and their related high social costs, various therapeutic strategies have been proposed for the treatment of spasticity including surgical, medical, and rehabilitation procedures. One of the major treatments for muscle tone modulation is botulinum toxin type A (BTX-A) administered intramuscularly [5,7]. BTX-A, by modulating the release of acetylcholine from synaptic vesicles, brings a reduction in muscle tone and, if associated with appropriate rehabilitation treatment, can stop the cascade of events that causes muscle fibrosis with subsequent retraction and joint blocks. BTX-A is indicated when spasticity is focal or segmental and interferes with active or passive operation. The primary purpose of spastic muscle treatment is to maintain length and to allow normal limb placement to avoid secondary shortening of soft tissues. In general, treatment with BTX-A is performed in addition to other rehabilitation strategies based on individualized multidisciplinary programs aimed at achieving tailored goals for each patient. Therapeutic plans must be precisely defined as they must balance the reduction of spastic hypertonia and retention of residual motor function. Although there is no consensus on when BTX-A therapy should begin or how long it should last, BTX-A intramuscular infiltrations are considered the first line of medical treatment for focal/segmental spasticity [8].

Proper use of botulinum toxin both in terms of dosage and injection requires careful monitoring of spastic hypertonia over time. The most commonly used muscular tone measurement is the Modified Ashworth Scale (MAS), where resistance to the passive muscle extension is rated in five points on an ordinal scale. MAS has been criticized for non-standardization of extension speed in manual trials, it does not quantify an absolute resistance, it is physician-dependent, only applies to distal body segments, and has low sensitivity to small variations in muscle tone [9]. We have also discussed the reliability and validity of this scale. MAS is reliable for measuring the muscle tone of some muscle groups such as the elbow, wrist, and knee flexors [10]. Considering the difficulties in distinguishing between the increase in muscle tone and soft tissue rigidity, as well as lack of correlation with functional changes after each treatment, appropriate clinical and instrumental measurements should be used to obtain reliable and accurate values of their rheological properties (tone, elasticity, and stiffness). A tool with these portable and reliable features is MyotonPRO^®^, a painless and non-invasive device which can provide quantitative and objective evaluations of muscle properties [11,12].

A proper assessment of muscle properties is important to make appropriate clinical decisions and to monitor therapy results in patients with spasticity.

The aim of this retrospective observational study is to objectify the efficacy and safety of 100 to 1000 units botulinum toxin treatment to modulate spasticity according to the individual patient’s needs.

## 2. Results

Patients were initially divided into three groups according to the botulinum toxin A dosage, as reported in the Methods section. During the observation period, some patients switched to another group because the dosage used increased (Table 1).

**Figure 1:**
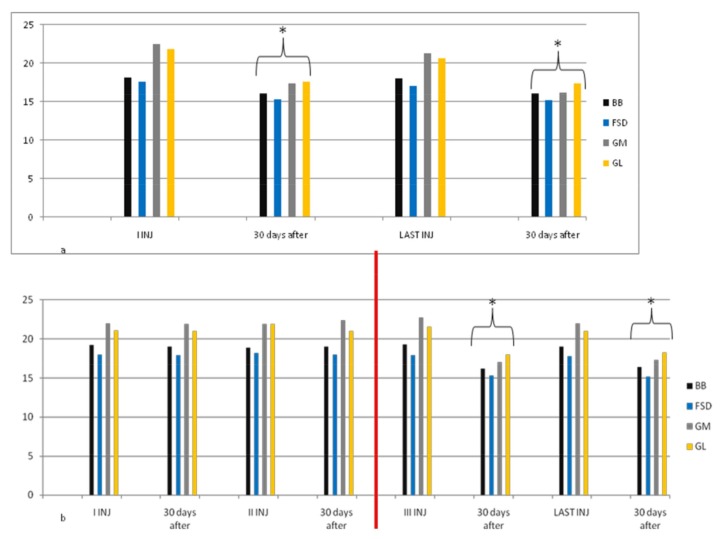
Myometric muscle tone evaluation in group A. This figure shows a statistically significant reduction in muscle tone after 30 days from the first and from the last injection in 20 patients treated with dosage up to 400 UI (**a**); For 10 patients of this group, muscle tone reduction was lower in the first and second cycle of injections; when we increased dosage (up to 700 UI, red line) due to increased units for each muscle or having injected other muscles not evaluated with myotonPRO^®^, reduction was statistically significant after 30 days for each cycle of injection (**b**). * *p* < 0.05. BB = Biceps Brachii, FSD = Flexorum Superficial Digitorum, GM = Gastrocnemius Medialis, GL = Gastrocnemius Lateralis.

**Figure 2:**
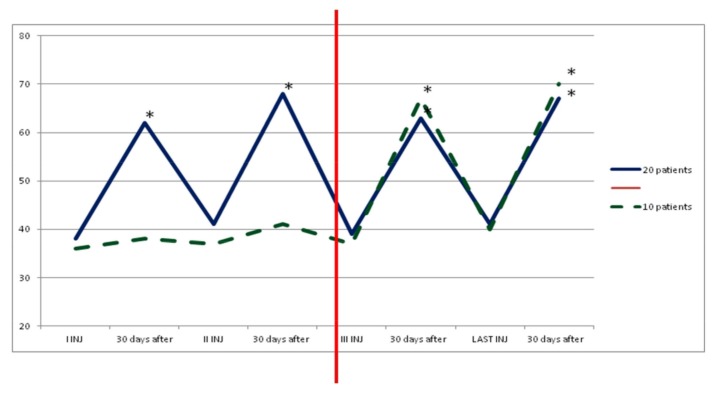
Functional Independence Measure (FIM) value in group A. The dark blue line shows statistical improvement of FIM in 20 patients treated with dosage up to 400 UI. Instead, the green and dashed line shows a statistical improvement of FIM in other 10 patients of this group when we increased dosage (up to 700 UI, red line) due to increased units for each muscle or to having injected other muscles. * *p* < 0.05. We considered the first three and last cycles of injection because statistical improvement of data occurred after dosage increased in 10 patients and it was constant until the end in both subgroups.

In group A, average doses increased during the study, but there was no statistical significance. At the third injection, dosage was increased in 10 patients (33%) due to poor clinical effects or to treat more muscles, so they switched to group B. In the following injections, these patients improved their clinical and instrumental measurement of spasticity (Figure 1 and Figure 2).

**Figure 3:**
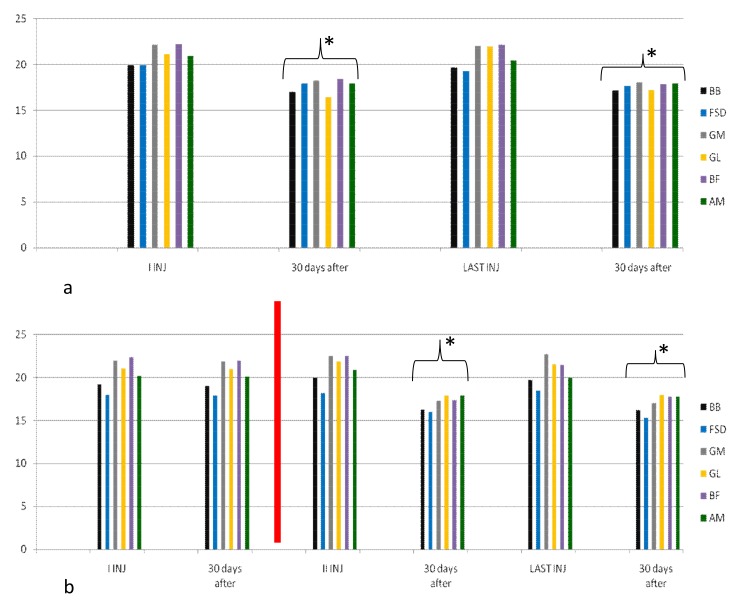
Myometric muscles tone evaluation in group B. This figure shows a statistically significant reduction in muscle tone after 30 days from the first and from the last injection in 32 patients treated with dosage from 400 UI to 700 UI (**a**); For 8 patients of this group, muscle tone reduction was lower in the first and second cycle of injections; when we increased dosage (up to 1000 UI, red line) due to increased units for each muscle (rather than having injected other muscles), reduction was statistically significant after 30 days for each cycle of injection (**b**). * *p* < 0.05. BF = Biceps Femoris, AM = Adductor Magnus.

**Figure 4:**
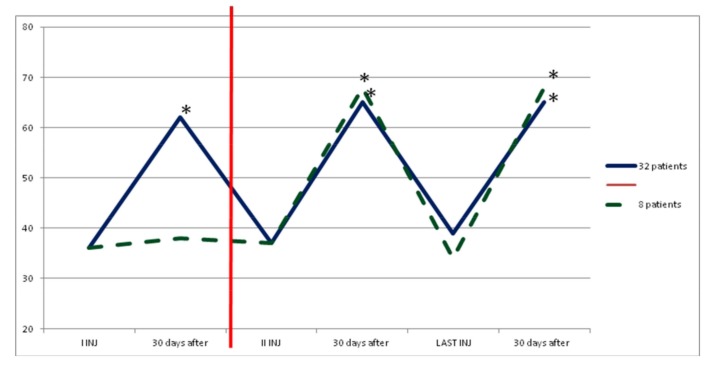
FIM value in group B. The dark blue line shows statistical improvement of FIM in 32 patients treated with dosage up to 700 UI. Instead, the green and dashed line shows a statistical improvement of FIM in other 10 patients of this group when we increased dosage (up to 1000 UI, red line) due to increased units for each muscle or having injected other muscles. * *p* < 0.05.

In group B, average increase in dosage was not statistically significant during the study. After the first injection, eight patients (20%) showed no clinical and instrumental improvement, so we increased dosage (we treated muscles with greater dosage rather than treating more muscles) and they switched to group C. In the following injections, these eight patients improved their clinical and instrumental measurement of spasticity (Figure 3 and Figure 4).

**Figure 5:**
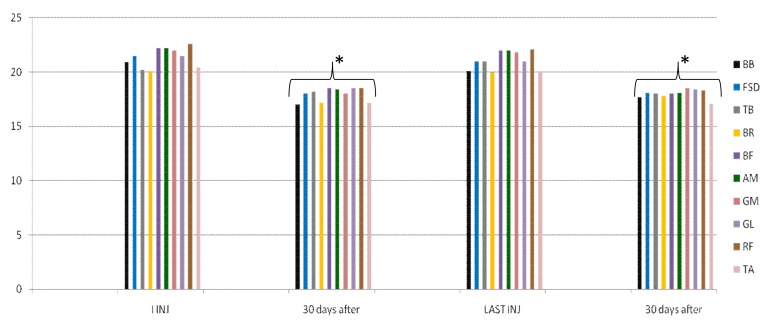
Myometric muscle tone evaluation in group C. In each cycle of treatment, a statistically significant reduction in muscle tone after 30 days from injection was found; this figure shows data of first and last cycle of treatment. * *p* < 0.05. TB = Triceps Brachii, BR = Brachioradialis, RF = Rectus Femoris, TA = Tibialis Anterior.

**Figure 6:**
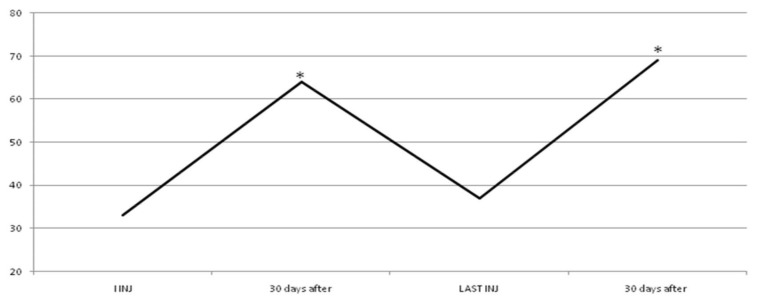
FIM value in group C. FIM value in all 50 patients treated with dosage from 700 UI to 1000 UI showed a statistically significant improvement (* *p* < 0.05). This figure shows values of first and last cycle of treatment.

In the group C, average doses at the end of study were statistically significant compared to the beginning (from 775.65 ± 30.45 to 986.65 ± 13.67, *p* <0.05). During the study all patients improved their clinical and instrumental measurement of spasticity (Figure 5 and Figure 6).

### Adverse Events

We found a good safety profile for long-term use of Incobotulinumtoxin A, also in patients treated with dosage up to 1000 UI. The adverse events reported were rare (Table 2).

Considering all injection sessions, only four cases (3.3%) of excessive local muscle weakness were found, two cases (1.6%) of transient generalized weakness lasting 20 and 10 days respectively and only one case of mild dysphagia (0.8%). More specifically:

- local muscle weakness: no case in group A, two cases in group B (5%) and two cases in group C (4%);
- transient generalized weakness: no cases in group A and B, two cases in group C (4%);
- mild dysphagia: no case in group A and B, one case in group C (2%; in the first cycle of injection, we also treated in this patient the left sternocleidomastoid muscle with 50 U of IncobotulinumtoxinA 1% saline due to muscle dystonia; this probably caused an adverse event because in the second cycle we treated the same muscle with 35 U of drug with good clinical response of dystonia and no dysphagia).

No patients abandoned treatment over the study.

## 3. Discussion

This study demonstrated long term treatment efficacy of IncobotulinumtoxinA in the management of muscle spasticity using variable doses.

According to the severity of spasticity, clinically and instrumentally evaluated, and to the number of muscles treated, we injected different botulinum toxin A doses measuring spasticity improvement after each injection cycle. In 10 of 30 patients in group A and 8 of 40 patients in group B, the dose administered was increased after the third and first injection respectively due to a non-significant clinical and instrumental effect; in group A, we increased units for each muscle or we increased number of muscles treated; in group B, we only increased units for each muscle. Different studies demonstrated that improper injection techniques or a denatured toxin resulted in therapeutic failure [13,14,15]. In the 18 patients of our study, injection technique or toxin A reconstitution were the same as the other patients, so therapeutic failure was due to incorrect dosage or clinical evaluation of the patient. After the total units administered were changed, spasticity improved after each injection in all of the 18 patients.

Changing dosage and using higher doses than approved is possible following a ratio known as Therapeutic Index (TI) or Therapeutic Ratio (TR) [16]. In clinical practice, the TI is the range of doses at which a medication appears to be effective in clinical trials for a median of participants without unacceptable adverse events. For most drugs, this range is wide enough, and the maximum concentration of the drug and the area under the concentration–time curve achieved when the recommended doses of a drug are prescribed lies sufficiently above the minimum therapeutic concentration and sufficiently below the toxic concentration [17]. Thus, it can be expected that at the recommended prescribed doses, drugs present clinical efficacy with an adequate safety margin. A higher TI means a safer drug. A drug is generally considered having a good safety profile if its TI exceeds the value of 10 [16,18]. Patients with multifocal spasticity require higher total doses of botulinum toxin A and several studies have demonstrated IncobotulinumtoxinA having a high TI. With escalating total doses, a higher number of spasticity patterns were successfully treated, leading to increasing improvements in muscle tone, shown by consistent decreases in clinical and instrumental evaluation. In this study, the use of doses from 100 to 1000 UI demonstrate that IncobotulinumtoxinA has a wide therapeutic window, indicating the drug has a good safety profile, since, even at high doses [13] side effects were mild and transient.

In this study we used myometric evaluation (Myoton PRO^®^) to objectively assess muscle tone. In an objective, simple, repeatable and non-invasive way, we evaluated even minimum changes in muscle tone; we obtained a broader pathophysiological vision of rheological properties of muscle tissue in different neurological, orthopedic, and sports pathologies [19]. In fact, during any pharmacological and/or physiokinesis and/or instrumental spasticity treatment, we studied therapeutic efficacy and we monitored the desired clinical effects without any alterations either in the omolateral antagonist muscles or in the contralateral agonists and antagonist muscles [20]. For example, wrist and finger control is very compromised after stroke; evaluating properties of the muscles responsible for these functions can help the physiatrist to realize the severity of the disability that the patient is encountering [21]. Instead, evaluation of triceps muscle tone may provide useful indications of load and balance alterations while standing and walking, with consequent repercussions on the entire musculoskeletal system [22,23].

In daily clinical practice, a number of organizational and methodological aspects must always be taken into account when planning a treatment strategy that includes the administration of botulinum toxin A [24]. These aspects include goals and treatment methods, clinical evaluation methods (MAS, myometry, FIM) injection programs (injecting muscles, injection technique, number of injection site for muscle, dose, and dilution), and other therapies (including targeted rehabilitation programs) to be integrated into the therapeutic plan [24,25]. Muscle weakness in the limbs and paresis is associated with spasticity and contributes to loss of motor dexterity and functional capacity. Spasticity, if untreated, can hinder the functional result by promoting persistent abnormal postures which produce muscular-tendon contractions and bone deformities [26]. Moreover, spasticity complications include movement impairment, difficulty in managing hygiene and self-care, poor self-esteem, body image alteration, pain, and pressure ulcers [26,27].

This study highlights the safety of high dose treatment with IncobotulinumtoxinA [28]. Few (less than 5%) and of no clinical relevance were the adverse events found. With different and high doses particularly, we obtained a safe and effective treatment for patients with chronic upper and lower limb spasticity following brain injury; using doses above 400 U enables treating a greater number of muscles and clinical spasticity patterns, resulting in increased improvements of muscle tone, goal attainment, and global efficacy, without compromising patients’ safety or tolerability [13,28]. High IncobotulinumtoxinA dosage offers the potential for comprehensive, well-tolerated and effective spasticity treatment of more clinical patterns, which allows greater focus on patients’ needs and goals with respect to lower doses in chronic spasticity [14,15,29].

Finally, our study also shows that repeated and long-term treatment (two years) with IncobotulinumtoxinA does not lead to any reduction in clinical efficacy due to antibodies forming against the active substance and/or excipients of the pharmacological preparation [30]. In fact, the possible development of botulinum toxin A antibodies related to injection frequency and dosage is a source of variability for possible adverse events [24]. In this study, several sets of high dose toxin exclude the adverse events and the development of antibodies against the toxin stimulated by higher doses. Certainly, using a highly purified botulinum toxin A formulation (IncobotulinumtoxinA), free from complexing proteins, is associated with a relatively low risk of immunogenicity and represents a therapeutic advantage for a long-term treatment with higher doses [13,14,15,29,30].

## 4. Conclusions

Botulinum toxin chemo-denervation has become popular because it is effective due to its local selectivity and its effects are repeatable and safe without important adverse events or the development of antibodies. To perform and optimize the treatment, an accurate clinical and instrumental evaluation of spasticity is needed to determine how this symptom is invalidating and to choose the best doses, muscles, and times of injection in each patient. Considering larger Therapeutic Index and Therapeutic Window (from 100 to 1000 UI) of IncobotulinumtoxinA, we can better modulate spasticity by considering its clinical significance for each patient. Therefore, we could introduce the concept of an “appropriate treatment” instead of a ”standard or high dosage treatment”; this allows us to underline the actual clinical needs of each patient.

## 5. Materials and Methods

This was a retrospective observational study lasting two years. One hundred and twenty adult patients (Table 1) with spasticity due to ischemic/hemorrhagic stroke, multiple sclerosis, spinal cord injuries, traumatic brain injury, and other cerebrovascular disorders were recruited. All subjects gave their informed consent for inclusion before participating in the study. The study was conducted in accordance with the Declaration of Helsinki, and the protocol number of the Ethics Committee was 5590. All patients signed the informed consent form. Exclusion criteria were: age above 80 or below 18, muscle fibrosis/tendon retraction detected by ultrasound, concomitant treatment with other muscle relaxants, peripheral myopathy/neuropathy, cognitive deterioration (Mini-Mental State Examination ≥ 24) and positive history of allergy to the drug.

### 5.1. Study Design

Patients were initially divided into three groups (data homogeneity are in Table 3) according to the botulinum toxin A dosage used at the beginning of study (IncobotulinumtoxinA, Xeomin^®^, Merz Pharma, 100 U/mL in normal saline):

(1) Group A (30 patients) up to 400 U
(2) Group B (40 patients) from 400 U to 700 U
(3) Group C (50 patients) from 700 U to 1000 U (maximum dose 600 U per limb).

The doses were chosen depending on the severity of spasticity clinically evaluated and on the number of muscles treated.

Treated muscles were Biceps Brachii, Brachioradialis, Triceps Brachii, Superficial FlexorumDigitorum, Ulnar FlexorumCarpis and Opponenspollicis for upper limb, Rectus Femoris, Biceps FemorisAdductor Magnus, Tibialis Anterior, Flexor Hallucis Longus, Gastrocnemius Medialis and Lateralis, Soleus, Tibialis Posterior and Flexor Digitorum for lower limb, with different average dosage (see Table 4 and Table 5).

During the study, patients received rehabilitation (stretching of injected muscles, active and passive limb mobilization, walking training, and global muscle strengthening) daily for the first 30 days after injection, then followed by three days a week until the next injection.

### 5.2. Outcome Measures

The evaluation method applied included the Functional Independence Measure (FIM, an international standard of disability measurement that differentiates motor from cognitive impairment; in our study we considered only motor impairment [31,32]) and myometric measurement (MyotonPRO^®^, tool that determines an objective value of muscle tone, elasticity and stiffness); furthermore, we took into consideration (muscle) tone values of superficial muscles [33,34]).

All assessments for each patient were performed at recruitment (during the 1st injection session), at every injection session, and during follow ups (one month after each session).

### 5.3. Statistical Analysis

Statistical analysis was carried out using the IBM SPSS Statistics program for Windows. Myometric measurements were analyzed with TWO WAY ANOVA method while FIM measurements with the t-student test. The alpha level for significance was set at *p* < 0.05. Data are expressed as average. In group A and group B we considered two patient subgroups according to dosage used at the beginning and the end of study.

## Figures and Tables

**Table 1:** Number of patients in each group at the beginning of study and after 9 months when there were no other switches. In the first group largest number of patients switched to another group (the second one).

Time	GR A 100–400 U	GR B 400–700 U	GR C 700–1000 U
t 0 (beginning)	30	40	50
t 1 (after 9 months)	20	42	58

**Table 2:** Summary of adverse events in each group. AE were rare and transient.

	1°GR 100–400 U BTX A	2°GR 400–700 U BTX A	3°GR 700–1000 U BTX A
Local muscle weakness	-	5%	4%
Transientgeneralized weakness	-	-	4%
Bradycardia	-	-	-
Dysphagia	-	-	2%
Dysphonia	-	-	-
Dyspnea	-	-	-
Constipation	-	-	-

**Table 3:** Patient demographics and baseline characteristics in the three groups; data demonstrate homogeneity of the samples.

	GR A 100–400 U BTX A	GR B 400–700 U BTX A	GR C 700–1000 U BTX A
N° patients	30	40	50
Age	64 ± 6.2	63 ± 8.4	66 ± 3.2
Sex M/F	17/13	23/17	28/22
Clinical: Hemip.dx	4	8	5
Hemip. sx	13	14	22
Monoparesis	4	1	0
Paraparesis	8	6	7
Tetraparesis	1	11	16

**Table 4:** This table shows how many patients of each group had a specific muscle treated and its percentage. In the first group, soleus was injected in 100% of patients; in the second group, the most treated muscle was biceps brachii, while in the third one they were triceps surae muscles.

Muscles Treated	GR A 100–400 U N° Patients	GR B 400–700 U N° Patients	GR C 700–1000 U N° Patients
Biceps brachii	28 93.3%	40 100%	43 86%
Brachioradialis	-	32 80%	41 82%
Triceps brachii	-	1 2.5%	38 76%
Superficial flexorumdigitorum	26 86.6%	34 85%	43 86%
Ulnar flexorumcarpis	-	22 55%	41 82%
Opponens pollicis	-	34 85%	43 86%
Rectus femoris	-	-	40 80%
Adductor magnus	-	26 65%	38 76%
Tibialis anterior	-	-	16 32%
Flexor alluci longus	-	18 45%	34 68%
Gastrocnemius medialis	27 90%	39 97.5%	50 100%
Gastrocnemius lateralis	27 90%	39 97.5%	50 100%
Soleus	30 100%	39 97.5%	50 100%
Tibialis posterior	-	26 65%	45 90%
Flexor digitorum brevis	-	16 40%	36 72%
Biceps Femoris	-	26 65%	36 72%

**Table 5:** Minimum, Maximum, Average ± SD dosage of each muscle in the three groups at the beginning of the study. In the first group, soleus received the highest dose compared to the other muscles (average 73.3 ± 2.5 UI). In the second group, muscles with highest doses were biceps brachii, superficial flexorum digitorum, gastrocnemius medialis, gastrocnemius lateralis, soleus, and tibialis posterior. In the third group, muscles with high doses were adductor magnus and those listed in the previous group.

Muscles	GR A 100–400 U Min-Max (Average ± SD)	GR B 400–700 U Min-Max (Average ± SD)	GR C 700–1000 U Min-Max (Average ± SD)
Biceps brachii	50–80 (60.2 ± 10.3)	70–90 (82.3 ± 10.1)	80–100 (91.2 ± 10.1)
Brachioradialis		50–60 (55.5 ± 10.4)	70–80 (75.3 ± 2.2)
Triceps brachii		50–60 (55.2 ± 10.1)	80–90 (85.6 ± 2.2)
Superficial flexorumdigitorum	50–80 (60.6 ± 10.2)	50–90 (75.3 ± 10.3)	100–150 (122.3 ± 20.1)
Ulnar flexorumcarpis		50–60 (55.5 ± 2.2)	80–100 (85.6 ± 2.1)
Opponenspollicis		20–30 (22.2 ± 10.3)	30–40 (33.4 ± 1.1)
Rectus femoris			70–80 (74.2 ± 1.1)
Adductor magnus		50–60 (55.3 ± 10.2)	100–150 (132.3 ± 20.2)
Tibialis anterior			50–60 (55.3 ± 2.4)
Flexor alluci longus		40–50 (44.2 ± 3.3)	50–60 (54.2 ± 1.3)
Gastrocnemius medialis	60–80 (73.4 ± 10.5)	80	100–150 (132.4 ± 10.4)
Gastrocnemius lateralis	60–80 (72.5 ± 10.4)	80	100–150 (122.4 ± 11.6)
Soleus	70–80 (76.3 ± 2.5)	80–90 (88.3 ± 1.3)	100–150 (135.7 ± 17.4)
Tibialis posterior		70–80(84.9 ± 3.3)	100–120 (111.2 ± 2.4)
Flexor digitorum brevis		50–60 (55.6 ± 3.4)	80–100 (89.9 ± 5.6)
Biceps Femoris		70–90 (84.6 ± 9.7)	100–150 (145.7 ± 14.9)
